# Revealing Criterial Vagueness in Inconsistencies

**DOI:** 10.1162/opmi_a_00025

**Published:** 2019-06

**Authors:** Steven Verheyen, Anne White, Paul Égré

**Affiliations:** 1 Laboratoire de Sciences Cognitives et Psycholinguistique, Département d’études cognitives, ENS, EHESS, PSL University, CNRS; 2 Laboratory for Experimental Psychology, Faculty of Psychology and Educational Sciences, KU Leuven; Laboratory for Experimental Psychology, Faculty of Psychology and Educational Sciences, KU Leuven; Institut Jean Nicod, Département d’études cognitives, ENS, EHESS, PSL University, CNRS

**Keywords:** categorization, vagueness, individual differences, semantic memory, ad hoc categories

## Abstract

Sixty undergraduate students made category membership decisions for each of 132 candidate exemplar-category name pairs (e.g., *chess* – Sports) in each of two separate sessions. They were frequently inconsistent from one session to the next, both for nominal categories such as Sports and Fish, and ad hoc categories such as Things You Rescue from a Burning House. A mixture model analysis revealed that several of these inconsistencies could be attributed to criterial vagueness: participants adopting different criteria for membership in the two sessions. This finding indicates that categorization is a probabilistic process, whereby the conditions for applying a category label are not invariant. Individuals have various functional meanings of nominal categories at their disposal and entertain competing goals for ad hoc categories.

## INTRODUCTION

In 2006 the number of planets in our solar system suddenly dropped from nine to eight. This dramatic change was not due to some astronomical catastrophe, but to a change in the criteria for Planets adopted by the International Astronomical Union (IAU). Seeing that Pluto has not cleared the neighborhood around its orbit as the new criteria prescribed, the IAU decided that Pluto should no longer be considered a Planet, but belongs in the category of Dwarf Planets. In 2015 the High Court of Tarbes (France) overruled the earlier decision by the supreme court of appeal (Cour de Cassation) that involuntary homicide cannot be committed on a fetus, effectively changing what it means to be a Person. Both examples serve to show that even in scientific and legal contexts, where precision is arguably of the utmost importance, concepts are vague and the criteria for determining whether an instance belongs in a category or not are subject to change (Egré, 2018). Most of the concepts we use in our daily lives can be argued to be vague.

In psychology, the vague rather than well-defined nature of categories was convincingly demonstrated by McCloskey and Glucksberg (1978), who showed that participants not only differed in opinion as to whether items should be considered category members or not, but also changed their answer when asked the same question one month later. Participants presented with a list of candidate instances for nominal categories such as Fish and Sports, responded with a nonmodal answer (a response that is different from the majority response) on 17% of the membership questions and provided inconsistent answers (a change in response after a one-month interval) on 12%. These results have been replicated by Hampton, Dubois, and Yeh (2006), who reported values for these inter- and intraindividual variability measures of 19% and 10%, respectively. Since the abandonment of the classic view of concepts as involving singly necessary and jointly sufficient membership conditions (Rosch, 1973; Rosch & Mervis, 1975; Ryle, 1949; Wittgenstein, 1953), these differences are not recognized as mistakes, but as a manifestation of faultless disagreement (Kölbel, 2004; Wright, 1995), or permissible variation (Raffman, 2014), indicating that there are multiple, equally competent ways of applying a vague concept.

Although the idea that nonmodal responses characterize vague categories was not new at the time (see Borel, 1907, and Black, 1937, for predecessors of the idea), McCloskey and Glucksberg’s (1978) work contributed to interindividual variability becoming a hallmark of vague categories. Nowadays, the existence of borderline items for which individuals can faultlessly disagree regarding category membership is considered to be central to what it means for a category to be vague (Kennedy, 2013; Smith, 2008; Wright, 1995).

The interindividual variability observed in categorization tasks is generally thought to result from both indeterminacy with respect to the conditions for application, and indeterminacy with respect to the extent of application given fixed conditions (Verheyen & Storms, 2013, 2018). Three people may disagree as to whether *chess* and *hiking* are Sports, because one believes Sports should have competitive and gamelike properties, while the other two only label activities that require physical effort Sports. On the basis of whether they consider *hiking* sufficiently effortful or not, the latter two could still disagree as to whether to call it a Sport. The former indeterminacy is commonly referred to as *criterial vagueness*, while the latter is known as *degree vagueness* (Devos, 1995, 2003; for a similar distinction, see Alston, 1964; Burks, 1946; Kennedy, 2013; Machina, 1976).

In contrast to interindividual variability, intraindividual variability has not caught on as a hallmark of vague categories. Although it has been acknowledged that vague categories have borderline cases for which an individual might feel equally inclined to apply and to deny the category label (Schiffer, 2003)—evidenced by increased categorization reaction times and lower confidence ratings (Koriat & Sorka, 2015), as well as competing responses to the same stimulus at a given time (Malt, 1990)—within-subject inconsistencies in categorization rarely constitute the topic of investigation themselves (see Hampton, Aina, Andersson, Mirza, & Parmar, 2012, for a notable exception). Intraindividual categorization differences tend to be accounted for in terms of shifting thresholds. What is believed to change from one occasion to the other, is the extent of the evidence the individual requires to apply the category label, not the conditions for application (Hampton, 1995; McCloskey & Glucksberg, 1978). Inconsistent answers are thus thought to reflect degree rather than criterial vagueness. The implicit assumption here seems to be that qualitatively different conceptions of a category might be entertained by different people, but not by an individual. It is this hypothesis that we put to the test in this article.

## OUTLINE

The observation by McCloskey and Glucksberg (1978) that people provide inconsistent answers when asked to repeat a categorization task indicates that the information that is retrieved from semantic memory is not invariant. The probabilistic nature of the semantic retrieval process is corroborated by the modest reliability of repeated exemplar generation (Bellezza, 1984a; White, Voorspoels, Storms, & Verheyen, 2014), category definitions (Barsalou, 1989; Bellezza, 1984b), feature importance ratings (Hampton & Passanisi, 2016), and typicality judgments (Barsalou, 1987, 1989; Hampton & Passanisi, 2016). While these studies allow one to establish *how much* change to expect from one occasion to the next, they do not indicate *what* it is that changes over time. The purpose of this article is to elucidate whether the criteria that are used to establish category membership may change.

Criterial vagueness has not yet been demonstrated within individuals. Different individuals have been shown to use distinct criteria for categorization, however (Verheyen & Storms, 2013; Verheyen, Voorspoels, & Storms, 2015; White, Storms, Malt, & Verheyen, 2018). This has been achieved using a mixture model that identifies subgroups of categorizers depending on the latent conditions they adhere to for categorization (criterial vagueness). Within each of the identified subgroups, the participants were also found to differ on the extent to which they required instances to demonstrate these conditions to be eligible for categorization (degree vagueness). The rationale behind the mixture model is that the use of distinct criteria will show in the relative frequency with which items are endorsed in subgroups. The items *chess* and *darts* will be more often categorized as Sports in a group emphasizing competitive and gamelike properties than in a group looking to physical exertion to establish category membership. The use of distinct thresholds will show in the proportion of categorized items. Participants who require little evidence for category membership will also include items that have relatively lower categorization frequencies in the subgroup, whereas very demanding participants will only include items that are frequently endorsed, as this indicates that these items score high on the subgroup’s categorization criterion.

Our design involves having participants complete a categorization task twice. We will apply the mixture model described above to the repeated data to determine whether any of the participants are assigned to different subgroups on the two occasions. This would indicate that their inconsistent answering reflects criterial vagueness. We will investigate both nominal categories like Fish and Sports, and ad hoc categories like Things You Rescue from a Burning House. Because unlike nominal categories, ad hoc categories violate the correlational structure of the environment and are not well established in memory (Barsalou, 1983), we expect more intraindividual categorization differences and more criterial accounts of inconsistent answers in ad hoc categories.

## DESIGN AND PROCEDURE

All materials, data, and analysis scripts are available on the Open Science Framework (Verheyen, White, & Égré, 2019a).

### Ethics Statement

This study was conducted with the approval of the Social and Societal Ethics Committee of KU Leuven. Written informed consent was obtained from all participants both at the start of the first and second categorization session.

### Participants

We invited 65 first-year psychology students at KU Leuven to take a categorization task twice, in exchange for course credit. Sixty of them completed both sessions (92%). Of these 60 participants, 5 were male (8.33%). The participants’ age ranged between 17 and 20 years (*M* = 18.08, *SD* = 0.65).

### Materials

Verheyen and Storms (2013) and Verheyen et al. (2015) investigated whether degree and criterial vagueness could account for interindividual categorization differences in nominal and ad hoc categories, respectively. We selected three nominal categories and three ad hoc categories from among the categories in these articles that showed evidence of criterial vagueness in the form of two subgroups of participants identified by the mixture analysis. Among the five qualifying ad hoc categories, we did not include the two categories with a very uneven distribution of participants over subgroups since we expected hardly any participants in our sample to subscribe to the categorization criteria of the smaller subgroup (comprising less than 10% of the participants in the original paper). In order to have an equal number of nominal categories, we randomly selected three among the four qualifying nominal categories.

The nominal categories Fish, Sports, and Tools had 24 items each. The ad hoc categories Things You Rescue from a Burning House, Means of Transport Between Brussels and London, and Weapons Used for Hunting had 20 items each. These items comprised the full range of category membership, including several clear members and clear nonmembers, but mainly borderline cases. All the materials were presented in Dutch.

### Procedure

Participants were administered a computerized categorization task in which the materials were presented in two blocks (nominal vs. ad hoc) of three categories each. The presentation order of blocks, categories within a block, and items within a category was randomized for every participant. Separate screens for each category would display the categorization instructions on top, indicating that participants could answer *yes* or *no* to the question of whether the items that followed belong to the category or not. A third response option, labeled *unknown*, was meant to be used when participants did not know a particular item or felt an item was ambiguous and did not know which meaning was intended.

Approximately one month after completing the categorization task, participants were presented the same task again. Following McCloskey and Glucksberg (1978), they were informed that some instances of the first session could appear again.

## RESULTS

We report the results in two separate sections. In the first, we use linear mixed-effects models to investigate whether the prevalence of inconsistencies across categorization sessions differs between nominal and ad hoc categories. In the second section, we apply the mixture model from the studies that informed the stimulus selection to the repeated categorization data in order to determine to what extent differences between sessions represent criterial vagueness.

### Prevalence of Inconsistencies

Seventy-three percent of the participants provided at least one inconsistent response (i.e., a change in response across sessions: *yes*/*no*, *yes*/*unknown*, or *no*/*unknown*) for each of the six categories. Sixteen participants (27%) answered consistently on one category, but not on the other five (Fish: *n* = 7; Rescue: *n* = 6; Sports, Transport, and Weapons: *n* = 1). No one answered consistently for the nominal category Tools. A parallel pattern was observed for the items. Inconsistent answers were observed for all items (*n* = 132) except for eight (94%). The items that yielded perfectly consistent answers were all clear members of the target categories (Fish: *goldfish*, *trout*; Sports: *skiing*, *swimming*, *tennis*; Tools: *axe*, *hammer*; Rescue: *people*). None of the items for the other two ad hoc categories yielded perfectly consistent answers.

**Figure 1.**
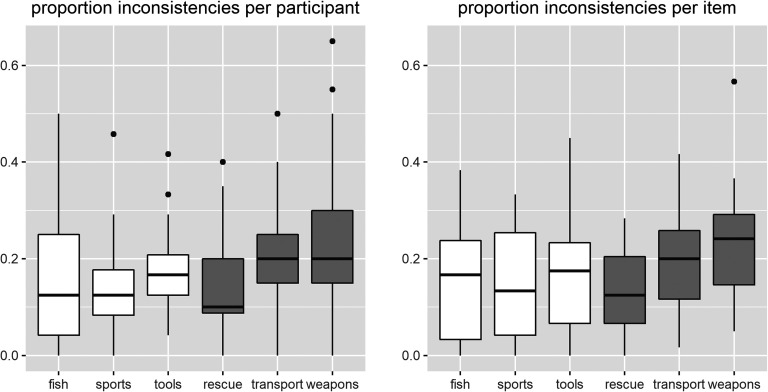
**Boxplots of the proportion of inconsistencies across sessions for each participant (left panel) and each target item (right panel).** Boxplots for nominal categories are depicted in white; boxplots for ad hoc categories are depicted in gray. The bands in the boxes represent the median values.

Figure 1 depicts the proportion of inconsistent responses for each of the participants (left panel) and each of the target items (right panel). On average, the participants provided an inconsistent answer on 16.06% of the membership questions for the nominal categories and on 19.00% of the membership questions for the ad hoc categories.

To establish whether ad hoc and nominal categories differed with respect to intraindividual differences, we determined whether participants’ repeated responses were inconsistent or not and fitted a binomial mixed effects model to the resulting variable, using the lme4 package (Bates, Maechler, Bolker, & Walker, 2015) in R version 3.4.3 (R Core Team, 2017). The fixed part of the model contained the main effect of the binary variable block, indicating whether the answers pertained to a nominal (1) or ad hoc (0) category. The random part of the model included random category, item, and participant intercepts, and an interaction between the block and participant variables. The main effect of block (*β* = −.35, *SE* = .19, *z* = −1.88, *p* = .06) was not significant at *α* = .05. This result was supported by comparing the BIC of the above model to that of an alternative model from which the main effect of block was removed (BIC_simple_ = 6,894.60 vs. BIC_full_ = 6,900.80).^1^ In other words, we did not reject the null hypothesis that the prevalence of intraindividual differences differs between nominal and ad hoc categories.

### Prevalence of Criterial Vagueness

**Figure 2.**
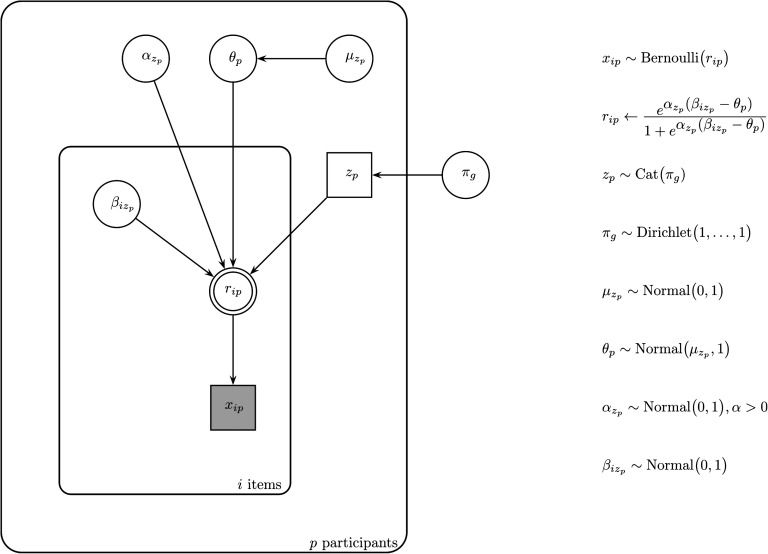
**Graphical model representation of the mixture model.**

Figure 2 holds a graphical depiction of the mixture model (Lee & Wagenmakers, 2014). It considers each categorization decision *x*_*ip*_ the outcome of a Bernoulli trial (1 for *yes*, 0 for *no*) with the probability of a membership response to item *i* by participant *p* expressed by *r*_*ip*_. It assumes the data result from a mixture of participants who adhere to different criteria for categorization. Depending on a participant’s latent group membership *z*_*p*_, different estimates are obtained for the item parameters *β*_*i*_, which express the extent to which the items display the group’s categorization criterion. The *β*_*i*_ are compared against the participant’s internal threshold *θ*_*p*_ to establish the items’ category membership. Whereas differences in *β*_*i*_ signal vagueness in criteria, differences in *θ*_*p*_ capture degree vagueness or the amount of evidence participants require for category membership. The parameter *α* determines for each group the shape of the function that relates the extent to which an item surpasses/falls short of the threshold to the probability of categorization. The function is S-shaped: it starts off at a zero when the *β*_*i*_ − *θ*_*p*_ difference is large and negative, demonstrates an increase for small differences between *β*_*i*_ and *θ*_*p*_, and asymptotes to one when the difference grows large and positive. The value of *α* reflects the steepness of the function at the point of subjective equality (the point for which the categorization probability equals .50, when *β*_*i*_ = *θ*_*p*_).

Latent group membership *z*_*p*_ is parameterized in the model as a categorically distributed random variable with *π*_*g*_ reflecting the probability of belonging to group *g*. The threshold parameters *θ*_*p*_ are drawn from normal hyper-distributions, parameterized by group-specific means and precision 1. We employed a uniform Dirichlet prior for the membership probabilities *π*_*g*_, a half-normal distribution centered at 0 with precision 1 for each *α*, and normal priors centered at 0 with the precision set to 1 for the remaining model parameters.

The repeated categorization data from the current study were merged with the categorization data that were available for the same materials from earlier work (Verheyen et al., 2015; Verheyen & Storms, 2013). The merging ensures that we have enough data to obtain reliable parameter estimates for the different subgroups. The two sets of categorization responses by the participants who took the categorization task twice were included as independent entries. *Unknown* responses were treated as missing values. For each of the nominal categories, the merged data thus comprised 370 categorization responses to each of 24 items (2 × 60 new responses + 250 responses from Verheyen & Storms, 2013). For the ad hoc categories, the merged data comprised 374 × 20 categorization responses (2 × 60 new + 254 from Verheyen et al., 2015). The mixture model was applied to these merged data sets.

Separate model estimates were obtained for each of the categories using WinBUGS (Lunn, Thomas, Best, & Spiegelhalter, 2000) by running three chains of 10,000 samples each, with a burn-in of 4,000 samples. The chains were checked for convergence and label switching. For every category a two-group solution was obtained since for all six selected categories two subgroups of participants were identified by the original mixture analyses (Verheyen & Storms, 2013; Verheyen et al., 2015). The original groups were recovered in the analysis of the merged categorization data, as evidenced by the correlations between the posterior means of the old and new group-specific *β*_*i*_ estimates (all *r* > .95). (For a substantive interpretation of the categorization criteria, see section 1 of the Supplemental Materials [Verheyen, White, & Égré, 2019b].)

The focus here will be whether the 60 participants who completed the categorization task twice are assigned to a different group upon repetition. This would indicate that they relied on distinct criteria for categorization in the two sessions. Group membership was determined based on the posterior mode of *z*_*p*_. We observed numerous group changes from Session 1 to Session 2.^2^ For only 27% of the participants no change in group membership was observed. These participants were placed in the same group on both occurrences of the categorization task for all six categories. Seventy-three percent of participants thus demonstrated a group change for at least one category. Thirty-two percent of participants changed group for two categories. Three percent changed group for three categories. There were no participants for which a group change was established for more than three categories. These percentages indicate that criterial vagueness is present *within* individuals.^3^

For the nominal category Fish, 9 out of 53 participants (17%) who demonstrated at least one inconsistency were placed in different groups on the two occasions. For Sports and Tools these percentages equaled 24% (14/59) and 28% (17/60), respectively. Fewer group changes were observed for the ad hoc categories: 11/54 (20%) for Rescue, 7/59 (12%) for Transport, and 9/59 (15%) for Weapons. We constructed a new variable indicating whether the mixture analysis placed participants in different groups on the two repetitions or not, and fitted a binomial mixed-effects model to it. The fixed part of the model contained the main effect of the binary variable block, indicating whether the answers pertained to a nominal (1) or ad hoc (0) category. The random part of the model included random category and participant intercepts, and an interaction between the block and participant variables. The effect of block (*β* = .48, *SE* = .28, *z* = 1.75, *p* = .08) was not significant at *α* = .05. This result was supported by comparing the BIC of the above model to that of an alternative model from which the main effect of block was removed (BIC_simple_ = 369.35 vs. BIC_full_ = 372.30). While group changes thus appeared less frequent for the ad hoc categories than for the nominal categories, this difference was not significant.

## GENERAL DISCUSSION

For three nominal and three ad hoc categories, participants decided on the category membership of various target items. They completed the task twice, separated by a one-month interval. Inconsistent answers were ubiquitous. Participants rarely provided identical responses on both occasions. We established that this intraindividual variability was not exclusively the result of degree vagueness (participants changing the amount of evidence required for membership, given constant conditions across sessions), as was assumed up until now. Several of these inconsistencies could be attributed to criterial vagueness: participants adopting different conditions for application in the two sessions. Each of these participants was placed in distinct groups on the two sessions by a mixture model that identifies latent groups of participants who employ different categorization criteria.

For nominal categories the existence of criteria differences within individuals indicates that people have various “meanings” at their disposal, which are probabilistically retrieved from semantic memory. McCloskey and Glucksberg (1978) refer to these meanings as *functional categories*, suggesting that they can be relied upon to serve different purposes (e.g., fish in the zoological vs. the seafood sense; see also Hampton et al., 2006, and Verheyen & Storms, 2013). The possibility to recruit different subsets of category knowledge allows for efficient processing in that information that is most relevant to accomplish particular tasks can be focused on (Yeh & Barsalou, 2006). It might make memories and truth judgments less reliable, however, as information recall and property verification might differ depending on the functional meaning that is accessed (Hampton et al., 2012). The challenge for future work is to determine how particular meanings are likely to become activated on a given occasion and to establish whether it is tenable to argue for context- and task-independent category representations if people are highlighting a particular conceptual content whenever they use a category label (Braisby, 1993).

The observation that inconsistent categorization responses can result from criterial vagueness holds for both nominal and ad hoc categories. We found no significant difference regarding the prevalence of inconsistencies or of criterial changes in nominal vs. ad hoc categories. This might strike some as surprising given that ad hoc categories are thought to be less rooted in the environment and in semantic memory than nominal categories are (Barsalou, 1983) and therefore might be expected to show less stability. The lack of a stability difference might be an indication that the ad hoc categories we selected should be considered goal-derived categories: ad hoc categories that have become well-established in memory, for example, through frequent use (Barsalou, 1985). The observation that one and the same individual may use different criteria for recruiting items that fulfill the category’s goal, would then be an indication that people sometimes entertain competing goals—such as traveling comfortably or fast between Brussels and London—the prominence of which might change from one occasion to the next (see also Voorspoels, Storms, & Vanpaemel, 2013, who showed that individuals can provide *multiple* ideal characteristics of goal-derived categories).

There is no reason to assume that the occurrence of criterial vagueness is specifically related to particular word classes (Verheyen & Storms, 2018). Our findings pertain to nominal and ad hoc categories, but are likely to generalize to other paradigmatic examples of vague categories, such as gradable adjectives like Intelligent and Healthy. The individual-level symptoms of vagueness that were discussed in the introduction for nouns have also been shown to exist for gradable adjectives. They too show competing responses to borderline items (borderline contradictions; see Alxatib & Pelletier, 2011; Egré & Zehr, 2018; Hersh & Caramazza, 1976; Ripley, 2011), increased reaction times and decreased confidence ratings for borderline items (Brownell & Caramazza, 1978; Hersh & Caramazza, 1976), and inconsistent responding across categorization sessions (Egré, de Gardelle, & Ripley, 2013; Hersh & Caramazza, 1976). Solt (2018) offers a treatment of how degree and criterial vagueness can account for inter- and intraindividual differences in the application of gradable adjectives. Much like Verheyen and Storms (2013) argued for nouns, she suggests that the judge- and context-dependent weighting of the multiple dimensions that underlie many gradable adjectives, is responsible for the observed variability in their use.

Our examples of vague concepts pertain to higher level categories, which tend to be comprised of heterogeneous instances that share similar functions rather than appearances. For perceptual categories it remains to be seen whether inconsistent answers can be attributed to criterial vagueness. Whether it can might depend on the frequency with which individuals categorize instances differently. We know that children as young as 14 months old can flexibly shift the criteria they use for categorizing objects in response to tasks requirements or instructions (for instance, from using shape to relying on material; Ellis & Oakes, 2006). We believe that the more this occurs, the more likely it becomes that individuals will develop multiple representations that remain accessible for later (functional) use (Schyns & Rodet, 1997).

Finally, this article advocates the study of intraindividual differences in vagueness research. Although interindividual variability is generally considered a hallmark of vague categories, its manifestation is not necessarily due to vagueness, but can be an indication of stable differences between subgroups of categorizers. For example, the same light stimulus may be categorized as one color by a color-normal perceiver, but stably (without uncertainty or unclarity being experienced) as another color by a person with protanopia (a form of color blindness characterized by a tendency to confuse reds and greens and by a loss of sensitivity to red light; Paramei, Bimler, & Cavonius, 1998). In addition, interindividual application differences can often be systematically related to properties of the individuals (tall people imposing higher height requirements than short people to name others tall; Verheyen, Dewil, & Egré, 2018; higher educated people applying nominal categories more conservatively; Verheyen & Storms, 2018; older people looking at traditional rather than modern materials to apply container labels; White et al., 2018). Intraindividual differences cannot be attributed to participants’ background differences and therefore provide a more direct window into the probabilistic nature of categories.

## ACKNOWLEDGMENTS

We thank the audience at ESSLLI 2017 for suggesting this study, and Tom Heyman for helpful comments on an earlier version of this article.

## FUNDING INFORMATION

SV was funded by the European Research Council under the European Union’s Seventh Framework Programme (FP/2007-2013) / ERC Grant Agreement 313610, and by KU Leuven Research Council grant C14/16032. AW is a Research Assistant at the Research Foundation–Flanders (FWO) and acknowledges KU Leuven Internal Research Fund PDM 18/084. PE was funded by ANR project TriLogMean (ANR-14-CE30-0010). SV and PE also acknowledge grants ANR-10-LABX-0087 IEC and ANR-10-IDEX-0001-02 PSL* for research conducted at the Department of Cognitive Studies of ENS in Paris.

## AUTHOR CONTRIBUTIONS

SV (Conceptualization: Equal; Data curation: Equal; Formal analysis: Lead; Methodology: Lead; Project administration: Equal; Software: Lead; Visualization: Lead; Writing – original draft: Lead; Writing – review & editing: Equal); AW (Data curation: Equal; Project administration: Equal; Resources: Lead; Writing – review & editing: Equal); PE (Conceptualization: Equal; Funding acquisition: Lead; Writing – review & editing: Equal).

## Notes

^1^ The smaller BIC value for the simple model indicates that it provides the more parsimonious account of the data in terms of fit and complexity (Schwarz, 1978). The increase in fit that is obtained by discerning between nominal and ad hoc categories in the full model does not warrant its added complexity/flexibility compared to the simpler model.^2^ Participants changing groups tended to have a high probability of being assigned to a subgroup in one session and a low probability of being assigned to the same group in the other session, rather than having similar assignment probabilities in both sessions. See section 2 of the Supplemental Materials (Verheyen et al., 2019b) for details.^3^ The percentages demonstrate that intraindividual variability can be due to criterial vagueness, but presumably overestimate the overall prevalence of criterial vagueness. We observed slightly more intraindividual categorization differences than earlier studies did (16% compared to 12% in McCloskey and Glucksberg, 1978, and 10% in Hampton et al., 2006). This discrepancy might be due to our selection of categories with known criterial vagueness (established between rather than within individuals). The earlier studies included a broader range of nominal categories than we did, which need not all display criterial vagueness. According to Verheyen and Storms (2013), 5 out of 8 categories in Hampton et al. (2006) demonstrated criterial vagueness; 6 out of 10 ad hoc categories in Verheyen et al. (2015) displayed criterial vagueness. The fact that we also counted an *unknown* response on one occasion and a *yes* or *no* response on the other occasion as inconsistencies might contribute to the discrepancy as well.
