# Supplementary material for: Revealing Criterial Vagueness in Inconsistencies
Source: Open Mind (Camb). 2019 Jun 1;3:41–51. doi: 10.1162/opmi_a_00025 (PMC6716385; doi:10.1162/opmi_a_00025)
Supplement: Supplementary file 1 [file opmi-03-41-s001.pdf]

# Revealing Criterial Vagueness in Inconsistencies: Supplementary Materials

Steven Verheyen, Anne White, & Paul Égré

## Substantive criteria differences

This section is intended to show that the group differences that have been identified through the mixture modeling approach correspond to substantive criteria differences.

In the mixture model the item parameters $\beta_i$ express the extent to which the items display the categorization criterion. The correlation between the group-specific item parameters therefore provides a first indication that the groups rely on different criteria. This correlation measures .76 for FISH, .89 for SPORTS, .89 for TOOLS, .84 for THINGS YOU RESCUE FROM A BURNING HOUSE, .54 for MEANS OF TRANSPORTS BETWEEN BRUSSELS AND LONDON, and .73 for WEAPONS USED FOR HUNTING. These correlations are high, but imperfect. They indicate that the participants in the different groups generally agree on the items that are appropriate category members and those that are not, but also signal that there are items for which the groups differ in opinion. This is exemplified in Figure 1, which shows for each category the proportion of participants from each of the identified groups who endorsed the various candidate items as category members. The categorization proportion in the group comprising the largest number of participants is depicted by gray squares. The categorization proportion in the group comprising the smallest number of participants is depicted by black circles. The items are ordered along the horizontal axes according to the categorization proportion in the larger of the two groups. For the larger group, this results in an S-shaped curve that is characteristic of vague categories (Egré, 2017; Hampton, 1998; Verheyen, Hampton, & Storms, 2010), with items that score low on the categorization criterion hardly ever being endorsed; a marked increase in categorization probability as items score higher on the categorization criterion; and items scoring the highest on the categorization criterion being almost invariable identified as category members.

If both groups were to rely on the same categorization criterion, we would expect the relative categorization order to be the same for both groups. In both groups we would expect to see a monotonic increase in categorization probability as items score higher on the common categorization criterion. That is, we would expect to see two S-shaped curves. This is clearly not the case in Figure 1. For each category one can identify items that are considered category members by the larger group of participants, but not by the smaller one, and vice versa. This indicates that the groups are not relying on the same criteria for categorization.

Figure 1 also contains for every item outlines of squares and circles, representing the posterior predictive distribution for the corresponding categorization probability in the larger and smaller group, respectively. The size of the outlines is proportional to the posterior mass that was given to the various categorization probabilities. The posterior predictive distribution represents the relative probability of different observable outcomes after the model has been fitted to the data and as such can be used to assess the fit of the model. Seeing that the posterior predictive distributions tend to be centered on the empirical categorization proportions, we can conclude that the mixture model fits the data. We can also see that there are several items for which the posterior predictive distributions are clearly different for the two groups, supporting the observation that the membership status of these items is regarded differently in the two groups.

The categorization patterns in Figure 1 also serve to show that the group differences are due to criteria differences (differences in the item parameters $\beta_i$) and not the result of

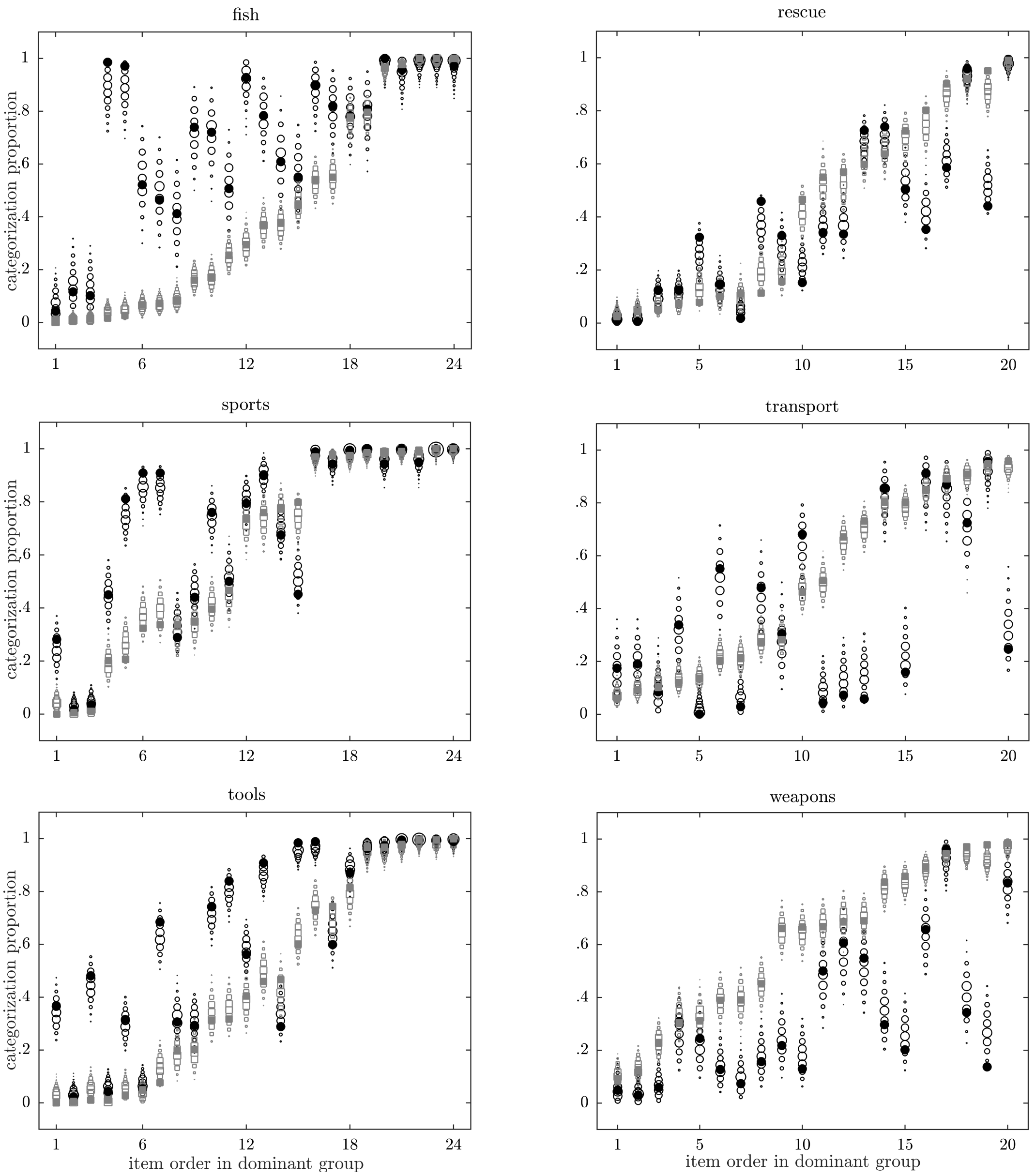


*Figure 1.* Posterior predictive distribution of the mixture model for the nominal (left) and ad hoc (right) categorization data. Filled gray squares show per item the categorization proportion in the larger group. Filled black circles show per item the categorization proportion in the smaller group. Items are ordered along the horizontal axes according to the categorization proportion in the larger group. Outlines of squares and circles represent the posterior predictive distributions of categorization decisions for the larger and smaller group, respectively. The size of these outlines is proportional to the posterior mass that is given to the various categorization probabilities.

differences pertaining to any of the other group-specific parameters in the mixture model. Were the groups identified based on differences regarding the group-specific mean thresholds $\mu$ or the group-specific $\alpha$'s, which determine the steepness of the function that relates the extent to which an item surpasses/falls short of the threshold to the probability of categorization, we would observe that the order of the categorization probability in the two latent groups would be the same. In the case of a group difference in $\mu$, we would observe two similarly shaped S-curves that are vertically displaced, indicating that on average one group applied a higher threshold to the same categorization criterion than the other group did. In the case of a group difference in $\alpha$, we would again expect the items to follow the same categorization probability order in the two groups, but one of the S-shaped curves to be steeper than the other. The observation that the categorization probability curves of the smaller of the two subgroups do not display an S-shape, similar to that of the larger groups, conveys that the categorization differences are due to group differences regarding $\beta_i$, not $\mu$ or $\alpha$. The group-specific mean parameter estimates for $\beta_i$, $\mu$, and $\alpha$ can be found on the project page on the Open Science Framework (`https://osf.io/mf7vq/`).

Further support for the claim that the group differences correspond to substantive criteria differences comes from a regression of the group-specific item parameters $\beta_i$ upon various participant-generated category attributes. In Verheyen and Storms (2013) and Verheyen, Voorspoels, and Storms (2015) the identified groups were shown to rely on different criteria for including items in a category. We conduct a similar analysis here for the $\beta_i$ estimates based on the merged data so that the reader can evaluate the claim of substantive criteria differences without having to work through the original papers.

Verheyen and Storms (2013) had participants generate attributes they considered important for something to be considered a member of the categories FISH, SPORTS, and TOOLS and had a different group of participants subsequently indicate whether the attributes that were generated at least twice applied to the various category items or not. A principal component analysis was then conducted on these applicability data, reducing the number of category attributes to 6 for FISH and SPORTS, and 7 for TOOLS. Voorspoels, Storms, and Vanpaemel (2013) employed a similar procedure for the ad hoc categories. They had one group of participants generate attributes that members of the ad hoc categories would ideally display and another group rate the degree to which the category items displayed these attributes on a 7-point Likert scale. Only attributes that were generated at least four times were included in the latter task, amounting to 9 attributes for THINGS YOU RESCUE FROM A BURNING HOUSE, 4 attributes for MEANS OF TRANSPORT BETWEEN BRUSSELS AND LONDON, and 13 attributes for WEAPONS USED FOR HUNTING.

For each of the identified subgroups we conducted a linear regression with forward selection procedure and a criterion of .05 to determine the category attributes that would enter the regression model. For the nominal categories, the group-specific item parameters $\beta_i$ were regressed upon the components obtained through the principal component analyses. For the ad hoc categories we regressed them upon the mean attribute ratings. Table 1 holds the results of the regressions analyses. For every group it shows the $R^2$ and the signs of the regression weights for attributes with a $p$-value less than .05. Attributes that did not contribute significantly are indicated by dots. The category attributes account well for the relative extent to which the items display the categorization criterion. The $R^2$ ranges between .54 and .96 with a mean of 85.92 ($SD = 12.10$) indicating that the item parameters

Table 1: $R^2$ and regression weights from the multiple regression analyses with forward selection procedure. The signs of the regression weights whose $p$-value was less than .05 are displayed, others are replaced by a dot. Group 1 always refers to the smaller of the two groups, while Group 2 refers to the larger of the two groups that were identied by the mixture model.

| | | | Category Attribute | | | | | | | | | | | | |
|---|---|---|---|---|---|---|---|---|---|---|---|---|---|---|---|
| Category | Group | $R^2$ | 1 | 2 | 3 | 4 | 5 | 6 | 7 | 8 | 9 | 10 | 11 | 12 | 13 |
| FISH | group 1 | .71 | - | - | . | . | . | . | | | | | | | |
| FISH | group 2 | .93 | - | . | . | - | + | . | | | | | | | |
| SPORTS | group 1 | .90 | - | + | . | - | . | + | | | | | | | |
| SPORTS | group 2 | .92 | - | + | . | . | . | . | | | | | | | |
| TOOLS | group 1 | .90 | - | + | . | . | . | . | . | | | | | | |
| TOOLS | group 2 | .94 | - | . | . | - | - | + | . | | | | | | |
| RESCUE | group 1 | .96 | + | + | . | . | . | . | . | . | + | | | | |
| RESCUE | group 2 | .90 | . | . | . | . | . | . | + | . | . | | | | |
| TRANSPORT | group 1 | .54 | . | . | + | . | | | | | | | | | |
| TRANSPORT | group 2 | .86 | . | + | . | . | | | | | | | | | |
| WEAPONS | group 1 | .82 | . | . | - | . | . | + | . | . | . | . | . | . | . |
| WEAPONS | group 2 | .93 | . | - | + | . | . | + | . | . | + | . | . | . | . |

$\beta_i$ can be related to substantive categorization criteria. The regression results also support our assertion that the categorization criteria differ from group to group in that different attributes predict the $\beta_i$ estimates of the different groups. This finding echoes the results in Verheyen and Storms (2013) and Verheyen et al. (2015), which was to be expected given the high correlations between the posterior means of the group-specific $\beta_i$ estimates in the current work and those in the original papers (all $\rho > .95$).

Note that this result also indicates that the differences between the groups are not the result of the items being assessed differently on the same criterion on the two test occasions. There is work demonstrating inter-individual inconsistency regarding the representation of exemplars: Young and older adults have been found to assess items differently on substantive criteria (Verheyen, Droeshout, & Storms, 2019). We do not know of any work that shows intra-individual inconsistency regarding the representation of exemplars, but we do not think it is responsible for the group differences we see here since the categorization criteria of the different groups can be related to different substantive attributes, suggesting that the individuals are indeed using distinct criteria on the different occasions.

## Nature of group changes

This section intends to show that for the participants who were found to switch groups/criteria, we could confidently discern the group membership on the two test sessions. It is based on the observation that participants changing groups tended to have a high probability of being assigned to a subgroup in one session and a low probability of being assigned to the same group in the other session, rather than having similar assignment probabilities in both sessions. The information in Table 2 pertains to the individuals who were found to change groups ($n$ out of 60 participants for every category). For every category the table lists summary statistics (mean $M$ and standard deviation $SD$) for the posterior mean of $z_p$, the categorical variable that does the group assignment in the mixture model. The information in the table is separated by the session in which the participants were assigned to the smaller subgroup (posterior mode of $z_p$ equals 0) vs. the larger subgroup (posterior mode of $z_p$ equals 1). The posterior mean of $z_p$ can be taken to express the relative evidence in favor of one group over the other. From Table 2 it becomes clear that the probability of being assigned to the same group differed considerably across sessions. For the category of FISH, for instance, the average posterior mean of $z_p$ was .11 on one occasion (clear evidence in favor of membership of the smaller group) and .99 on the other occasion (clear evidence in favor of membership of the larger group). Across categories, these values average .15 and .86.

Table 2: Mean and standard deviation of the posterior mean of $z_p$ across participants changing groups separated by the session in which they were assigned to the smaller vs. the larger subgroup based on the posterior mode of $z_p$.

| | | $mo(z_p) = 0$ | | $mo(z_p) = 1$ | |
|---|---|---|---|---|---|
| Category | $n$ | $M$ | $SD$ | $M$ | $SD$ |
| FISH | 8 | .11 | .16 | .99 | .01 |
| SPORTS | 14 | .14 | .12 | .83 | .17 |
| TOOLS | 17 | .19 | .18 | .87 | .13 |
| RESCUE | 11 | .22 | .15 | .66 | .14 |
| TRANSPORT | 7 | .08 | .16 | .95 | .10 |
| WEAPONS | 9 | .12 | .15 | .93 | .06 |

Figure 2 conveys the information in Table 2 in another way. The posterior means of $z_p$ of all the participants who were found to change groups are displayed in a histogram, which suggests that the values display a bimodal distribution. To confirm this, we conducted two-group normal mixture modeling (Scrucca, Fop, Murphy, & Raftery, 2016) on these values for every category. In Figure 2 the posterior means of $z_p$ are color-coded according to their group assignment by the two-group normal mixture model. These analyses again show that the group changes were rather evident. Posterior means of $z_p$ favoring membership of the smaller group ($z_p < .50$ corresponding to a posterior mode of 0) were clearly discerned from the posterior means of $z_p$ favoring membership of the larger group ($z_p > .50$ corresponding to a posterior mode of 1). Only in 7.58% of the participants changing groups, was the

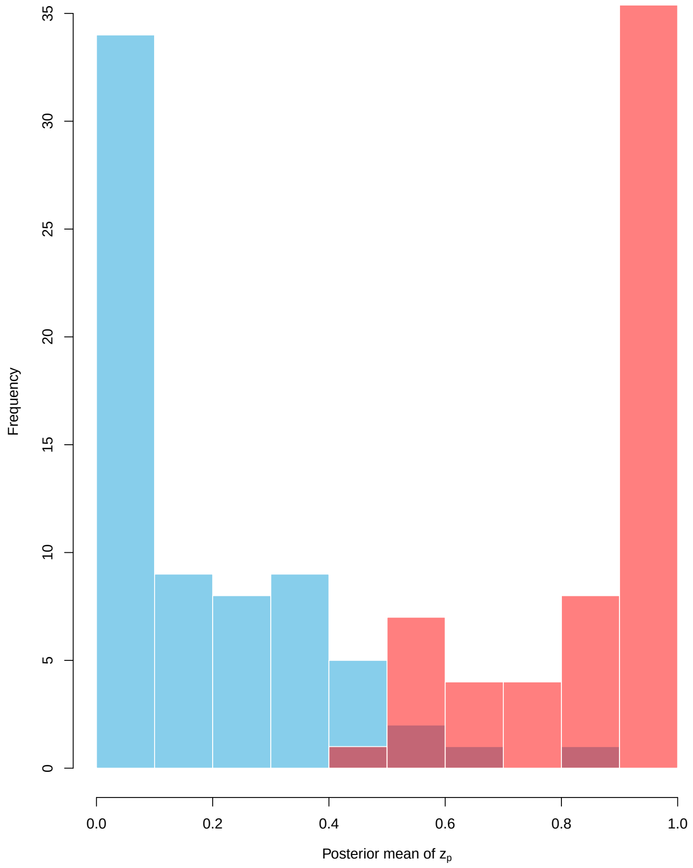


*Figure 2.* Histogram of the posterior means of $z_p$ of all the participants who were found to change groups, color-coded according to their group assignment in two-group normal mixture modeling intended to show the bimodality of $z_p$.

posterior mean of $z_p$ for session 1 and 2 not differentiated in the two-group normal mixture modeling. For the category THINGS YOU RESCUE FROM A BURNING HOUSE, for instance, the posterior means of $z_p$ of one participant were .496 and .510, which were not differentiated in the two-group normal mixture modeling. (The overlapping entries in Figure 2 signal the conflicting values, with the far left one corresponding to the .496 value from the example being included with the instances with a relatively high value for $z_p$.) The two-group normal mixture modeling thus confirms the bimodality of $z_p$, the parameter that does the group assignment. Only for 5 out of 66 participants who were identified to change groups was the group assignment somewhat uncertain in that these participants had similar (intermediate) assignment probabilities in both sessions. The large majority of the participants changing groups, however, tended to have a high probability of being assigned to a subgroup in one session and a low probability of being assigned to the same group in the other session.

# References


Egré, P. (2017). Vague judgment: A probabilistic account. *Synthese*, *194*, 3837-3865.

Hampton, J. A. (1998). Similarity-based categorization and fuzziness of natural categories. *Cognition*, *65*, 137-165.

Scrucca, L., Fop, M., Murphy, T. B., & Raftery, A. E. (2016). mclust 5: Clustering, classification and density estimation using gaussian finite mixture models. *The R Journal*, *8*, 205-233.

Verheyen, S., Droeshout, E., & Storms, G. (2019). *Age-related degree and criteria differences in semantic categorization.* Manuscript submitted for publication.

Verheyen, S., Hampton, J. A., & Storms, G. (2010). A probabilistic threshold model: Analyzing semantic categorization data with the Rasch model. *Acta Psychologica*, *135*, 216-225.

Verheyen, S., & Storms, G. (2013). A mixture approach to vagueness and ambiguity. *PLoS ONE*, *8(5)*, e63507.

Verheyen, S., Voorspoels, W., & Storms, G. (2015). Inferring choice criteria with mixture IRT models: A demonstration using ad hoc and goal-derived categories. *Judgment and Decision Making*, *10*, 97-114.

Voorspoels, W., Storms, G., & Vanpaemel, W. (2013). Similarity and idealness in goal-derived categories. *Memory & Cognition*, *41*, 312-327.
